# Exposure of bakery and pastry apprentices to airborne flour dust using PM_2.5 _and PM_10 _personal samplers

**DOI:** 10.1186/1471-2458-7-311

**Published:** 2007-11-01

**Authors:** Estelle Mounier-Geyssant, Jean-François Barthélemy, Lory Mouchot, Christophe Paris, Denis Zmirou-Navier

**Affiliations:** 1 INSERM (National Institute of Health and Medical Research) Research unit ERI n°11, 9 avenue de la Forêt de Haye, BP 184, 54505 Vandoeuvre-lès-Nancy, Cedex, France; 2 School of Medicine, Nancy University Medical School, 9 avenue de la Forêt de Haye, BP 184, 54505 Vandoeuvre-lès-Nancy, Cedex, France; 3 LICE-INRS, Department of exposure assessment, avenue de Bourgogne, 54500 Vandoeuvre-lès-Nancy, France

## Abstract

**Background:**

This study describes exposure levels of bakery and pastry apprentices to flour dust, a known risk factor of occupational asthma.

**Methods:**

Questionnaires on work activity were completed by 286 students. Among them, 34 performed a series of two personal exposure measurements using a PM_2.5 _and PM_10 _personal sampler during a complete work shift, one during a cold ("winter") period, and the other during a hot ("summer") period.

**Results:**

Bakery apprentices experience greater average PM_2.5 _and PM_10 _exposures than pastry apprentices (p < 0.006). Exposure values for both particulate fractions are greater in winter (average PM_10 _values among bakers = 1.10 mg.m^-3 ^[standard deviation: 0.83]) than in summer (0.63 mg.m^-3 ^[0.36]). While complying with current European occupational limit values, these exposures exceed the ACGIH recommendations set to prevent sensitization to flour dust (0.5 mg.m^-3^). Over half the facilities had no ventilation system.

**Conclusion:**

Young bakery apprentices incur substantial exposure to known airways allergens, a situation that might elicit early induction of airways inflammation.

## Background

Bakers and pastry cooks are exposed to flour dust and associated aeroallergen during the process of flour manipulation [1-3]. This may elicit airways sensitization and trigger the chain of events eventually leading to occupational asthma. A variety of respiratory effects have been described among bakers, including impairment of pulmonary function and chronic bronchitis [1,4-11]. Bakery is described as an occupation at risk of asthma [6,12-14] and ranks first, in France, based on data from the national observatory of occupational asthma [15].

Exposure to flour dust of bakers, pastry-makers and mills workers is well documented. For instance, in 1989, Musk *et al *measured total dust workplace levels among doughmakers and oven staff and described average values of 2.7 mg.m^-3 ^and 1.7 mg.m^-3 ^respectively, extreme figures reaching up to 14.1 and 37.6 mg.m^-3 ^[1]. Jauhiainen measured total dust workplace concentrations among subjects making dough (average concentrations of 4.6 mg/m^3^, ranging [0.9–14.7]) or making bread (2.3 [1.5–3.5] mg.m^-3^) [2]. In a recent study, Bulat [16] measured average concentrations of 2.1 [0,3–13,3] mg.m^-3 ^during a complete shift among bakers [16]. Inhalable dust concentrations among workers whose tasks were described as "weighing and mixing", and "making dough" were in the same order of magnitude (4.7 mg.m^-3 ^(average concentrations) and 3.3 mg.m^-3^) [17].

By contrast, there is a lack of data regarding exposure among bakery or pastry apprentices, although respiratory conditions have been described in this young population [18-24]. In the framework of a prospective study aiming at assessing how non asthmatic bakery and pastry apprentices may develop airway inflammation early in the course of their training, an exposure study was undertaken to assess personal exposure to flour dust and to describe the tasks that involve contact with flour in the bread making and pastry confectioning processes.

## Methods

With the collaboration of a large apprenticeship school in Nancy, Lorraine region, in NorthEast of France, we conducted a cross-sectional study in different classes of bakery and pastry training programmes during the 2003–2004 and 2004–2005 academic years. The study involved two stages. First, all apprentices following the first or second year of their training programme completed in their classroom (with 20 to 30 apprentices per classroom) a questionnaire describing their work environment and activities. In the second stage, volunteers were asked to participate to personal exposure measurements while at work. Agreement of the bakers and pastry confectioners who trained the volunteering apprentices was also requested. Typically, an apprentice works 3 weeks in the bakery or pastry shop and attends school for practical and academic classes during one week. But for special reasons (such as being fired or because of resignation) an apprentice stays in the same bakery or pastry shop until he/she graduates. The field exposure measurement study was conducted during a regular workday, most of the time between Monday and Friday, depending when bakery or pastry shop owners were available.

### Completion of questionnaires

The questionnaire was designed after a literature review, observations in several bakeries and following visits during the practical training classes in the apprenticeship school. It was validated by the school instructors and by heads of the local and regional bakery and pastry federations. A pilot study was undertaken during academic year 2002–2003 to check suitability of the questions for the study population. Information of interest encompassed personal characteristics, duration of a work shift, spatial organization of the bakeries or pastry shops, and ventilation characteristics and equipments (automatic or manual organization). The questionnaire also requested students to describe in detail their activities during a "standard" workday. Questionnaire data were input with Epidata software. Statistical analysis was carried out using the SAS statistical package and Statgraphic.

### Field exposure measurements and determination of flour dust concentrations

Volunteers for the exposure study were mostly recruited among second graders because younger apprentices were expected to have little technical activities during the first year of training. Further, their enrolment in the school spans over several months and they may change of practice location during the first months of their training programme. After they had declared their willingness to participate to this second stage of the study, during completing of the first phase questionnaire, names and phone numbers of bakery and pastry shop owners or managers were noted, in order for the study coordinator to request authorization to undertake the measurement study in the bakery or pastry shop. Study subjects were given a letter explaining the aim of study and providing general information on asthma and occupational hygiene. Apprentices were asked to participate during two series of personal exposure measurement runs, one during the "cold season", from February to April, and the second during the "hot season", from April to August, in order to asses seasonal variations.

To measure flour dust personal exposures, both PM_2.5 _and PM_10 _fractions were collected using the Harvard Chempass sampler [25] connected to a portable BGI pump (model 400) providing an air flow of 1.8 l/min in each sampling head. Measurements were done during a complete 5 to 11 hours work shift, volunteers carrying the sampler installed in a rucksack; the sampling heads were near the breathing zone. Field control filters were also placed in some rucksacks and processed along with those that collected flour dust. After shift, all filters were brought back within one hour to the laboratory and stored in a refrigerator (+/- 4°C) with a dessicator. Particles of flour dust were collected on Teflon filters (Gelman^®^, diameter 37 mm, porosity 2 μm) that were weighted using a microbalance (Mettler AT 261) with a sensitivity of +/- 0.01 mg. The balance was first calibrated and zeroed before each series of weight. Electrostatic charges on filters were eliminated using a deionization system. Filters were weighed prior to field sampling and after being loaded with flour dust. Controls were also included in the filters batches to be weighed; these batches were composed of 6 flour dust, 4 field control and 10 laboratory control filters. The day prior weighing, filters were retrieved from the refrigerator and kept in a room controlled for temperature (20 +/- 2°C) and humidity (33 +/- 4 % relative humidity). Seasonal variations of exposures were assessed for PM_2.5 _and PM_10 _over all bakers and pastry makers, with paired and signed rank tests. Median exposure values between apprentices' groups were compared over the two study seasons with Wilcoxon Mann-Whitney tests, because conditions for parametric tests were not met. However, for the sake of comparability with the literature data, where average values are usually shown, we also exhibit means and standard deviations in the text and table.

## Results

All apprentices completed the questionnaires (N = 286). Among them, not all participated to the personal exposure study. Among the 198 apprentices engaged in the second year of their training programme, 58 accepted to contribute to exposure measurements, but 24 employers declined participation; so 34 measurements were done during the cold season and 21 in summer, because of final school exams and tight schedule constraints (Table 1).

**Table 1:** Description of the study population

Study population (questionnaire study and exposure measurement study)						
	All apprentices (n = 286)^a^	Training level	Apprentices solicited for the exposure measurement study	Volunteers/Employers who accepted	Volunteers for exposure measurement study	
					
					Cold Season	Hot season

Bakers apprentices	128	38^b^/90^c^	90 ^c^	37/21	21	17
Pastry apprentices	158	50^b^/108^c^	108 ^c^	21/13	13	4

a : number of questionnaires completed

b : first year

c : second graders only

### Work environment

Table 2 shows the distribution of time spent at work according to the training level and indicates, as expected, longer shifts declared by second graders, especially among bakery apprentices. While all pastry apprentices worked in craft facilities (pastry or combined pastry-bakery), this was the case for 84% of bakery apprentices, 16% being in large bakery-pastry plants). The magnitude of flour handling is greater in craft production conditions.

**Table 2:** Duration of daily shift declared by apprentices, according to training level

Training category and level		[4 h – 8 h[ (%)	[8 h – 10 h[ (%)	[10 h – 12 h](%)
Bakery apprentices	First year (n = 38)	31 (81.7)	5 (13.1)	2 (5.2)
	Second year (n = 90)	49 (54.5)	37 (41.1)	4 (4.4)
Pastry apprentices	First year (n = 50)	35 (70.0)	14 (28.0)	1 (2.0)
	Second year (n = 108)	69 (63.9)	35 (32.4)	4 (3.7)

More than 1 out of 3 bakery apprentices (4/5 among pastry apprentices) declared absence of ventilation, be it natural or mechanical, or of air conditioning. For 16% of apprentices, the room where he/she spent most of the time did not have a door opening outside, and 24 % had no window.

Tasks associated with manipulation of flour dust and cleaning activities involving exposure to dust according to the literature are described in Table 3 and 4 which exhibit the frequency distribution of times these tasks are accomplished during a typical day, as reported by the questionnaires. Data are split according to training level; also are exhibited (last column) the answers given by the subgroup of apprentices who volunteered to participate to the exposure measurements. The tasks varied markedly between the two groups (bakers or pastry) according to training year. Second graders, among pastry apprentices, are more engaged than younger students in tasks involving exposure to flour dust (mixing, dividing and shaping dough into pieces, moulding dough pieces; all p values ≤ 0.02). By contrast, tasks are more evenly distributed among the first training year for bakers, only flour weighing for pastry production being more frequent among younger bakery apprentices (75 respondents, p = 0.03).

**Table 3:** Daily frequencies of activities associated with manipulation of flour dust for bread and pastry production

		**Bakery apprentices**		**Pastry apprentices**		**Volunteers**	
		**First year**	**Second year**	**First year**	**Second year**	**Bakery apprentices**	**Pastry apprentices**
		
		(n = 38)^a^	(n = 90)^a^	(n = 50)^a^	(n = 108)^a^	(n = 21)^a^	(n = 13)^a^
		(max = 37)^b^	(max = 83)^b^	(max = 44)^b^	(max = 80)^b^	(max = 20)^b^	(max = 11)^b^
		(min = 35) ^c^	(min = 40)^c^	(min = 40)^c^	(min = 44)^c^	(min = 7)^c^	(min = 8)^c^

Weighting flour for bread production	0 per day	16.7*	15.2	75.0	84.1	0.0	88.9
	[1 – 3]	58.3	45.7	20.5	11.4	70.0	11.1
	[4 – 15]	25.0	39.1	4.5	4.5	30.0	0.0
Weighting flour for pastry production	0 per day	63.9	84.6	17.4	17.4	100.0	25.0
	[1 – 3]	25.0	15.4	45.6	37.0	0.0	25.0
	[4 – 15]	11.1	0.0	37.0	45.6	0.0	50.0
Loading flour in mixer	0 per day	25.0	11.4	83.3	73.1	10.0	81.8
	[1 – 3]	52.8	59.5	16.7	24.4	70.0	18.2
	[4 – 10]	22.2	29.1	0.0	2.5	20.0	0.0
Mixing	0 per day	21.6	9.0	83.7	66.2	11.1	90.0
	[1 – 3]	46.0	55.0	14.0	27.3	66.7	10.0
	[4 – 10]	32.4	36.0	2.3	6.5	22.2	0.0
Dividing and shaping dough into pieces (manual or automatic)	0 per day	8.1	10.0	83.7	61.0	5.3	90.0
	[1 – 2]	46.0	42.5	11.6	28.6	63.1	10.0
	[3 – 10]	43.2	45.0	4.7	10.4	26.3	0.0
	[11 – 20]	2.7	2.5	0.0	0.0	5.3	0.0
Moulding dough pieces (manual or automatic)	0 per day	14.3	4.8	88.1	69.3	5.3	90.0
	[1 – 2]	42.8	51.8	11.9	20.0	63.1	0.0
	[3 -5]	34.3	31.3	0.0	8.0	26.3	10.0
	[6 – 20]	8.6	12.1	0.0	2.7	5.3	0.0

a: numbers apprentices by level, b: maximum number of respondents for the set of questions, c: minimum number of respondents for the set of questions. * In % of respondents.

**Table 4:** Daily frequency of cleaning activities associated with manipulation or presence of flour dust

		**Bakery apprentices**		**Pastry apprentices**		**Volunteers**	
		**First year**	**Second year**	**First year**	**Second year**	**Bakery apprentices**	**Pastry apprentices**
		
		(n = 38)^a^	(n = 90)^a^	(n = 50)^a^	(n = 108)^a^	(n = 21)^a^	(n = 13)^a^
		(max = 37)^b^	(max = 85)^b^	(max = 49)^b^	(max = 100)^b^	(max = 21)^b^	(max = 12)^b^
		(min = 34)^c^	(min = 64)^c^	(min = 47)^c^	(min = 74)^c^	(min = 19)^c^	(min = 10)^c^

Cleaning of tables for bread and pastry production	0 per day	2.8*	3.6	4.1	1.0	4.8	0.0
	[1 – 3]	69.4	72.6	37.5	39.0	71.4	33.3
	[4 – 5]	16.7	14.3	27.1	13.0	14.3	0.0
	[6–40]	11.1	9.5	31.3	47.0	9.5	66.7
Cleaning floor	0 per day	10.8	8.2	6.1	4.1	5.3	9.1
	1	78.4	78.8	87.8	85.7	84.2	72.7
	[2 – 6]	10.8	13.0	6.1	10.2	10.5	18.2
Cleaning oven (sweeping of burned dust)	0 per day	27.8	18.7	77.3	75.6	21.1	90.0
	1	47.2	46.7	15.9	18.0	36.8	0.0
	[2 – 5]	19.4	29.3	6.8	5.1	42.1	0.0
	[6 – 15]	5.6	5.3	0.0	1.3	0.0	10.0

a: numbers apprentices by level, b: maximum number of respondents for the set of questions, c: minimum number of respondents for the set of questions. * In % of respondents.

### Personal exposure measurements

Table 5 presents personal exposure levels to flour dust for bakery and pastry apprentices, according to the particles fraction, respectively during the hot and cold seasons. Average (and standard deviate) personal exposures to PM_2.5 _for bakery apprentices are respectively 0.50 [0.37] mg.m^-3 ^during the hot season and 0.71 [0.37] mg.m^-3 ^during the cold season (corresponding values for pastry apprentices are 0.29 [0.06] mg.m^-3 ^and 0.35 [0.17] mg.m^-3^). Exposure values are greater during the cold sampling period over the two apprentices categories (p = 0.009; 21 measurements with both seasonal values). PM_10 _personal exposures for bakers are 0.63 [0.36] mg.m^-3 ^in summer and 1.10 [0.83] mg.m^-3 ^in winter. Corresponding exposures are respectively 0.47 [0.13] mg.m^-3 ^and 0.44 [0.16] mg.m^-3 ^among pastry apprentices. Again, winter figures are greater over the two apprentices categories (p = 0.003, based on 17 measurements).

**Table 5:** Personal exposures levels to flour dust of apprentices in bakeries and pastry shops

	**PM_2.5_**		**PM_10_**	
	
	**Summer**	**Winter**	**Summer**	**Winter**
**Baker apprentices**	(n = 17)^a^	(n = 21)	(n = 15)	(n = 20)
	0.50 ^b ^(0.37) ^c^	0.71 (0.37)	0.63 (0.36)	1.10 (0.83)
	[0.17–1.52] ^d^	[0.19–1.42]	[0.17–1.73]	[0.28–4.04]
**Pastry apprentices**	(n = 4)	(n = 13)	(n = 3)	(n = 13)
	0.29 (0.06)	0.35 (0.17)	0.47 (0.13)	0.44 (0.16)
	[0.22–0.36]	[0.20–0.70]	[0.33–0.58]	[0.22–0.82]

a: Each cell comprises the number of samples, b: the arithmetic mean, c: standard deviate, d: [min-max] (in mg/m^3^).

Average exposures of bakery and pastry apprentices over the two seasons are respectively 0.61 [0.38] mg.m^-3 ^and 0.32 [0.15] mg.m^-3 ^for PM_2.5 _(corresponding PM_10 _values are 0.87 [0.70] mg.m^-3 ^and 0.46 [0.15] mg.m^-3^).Median values for PM_2.5 _(p = 0.006) and PM_10 _(p = 0.001) were significantly greater among bakers.

To assess whether seasonal variations of humidity could have altered filters weight comparisons, differences of pre and post sampling weights were compared between the two study periods and showed similar patterns (n = 80 ; p = 0.96).

## Discussion

According to published data, average exposure levels (inhalable or total dust) for weighing, mixing, dividing, moulding, dough and bread making, and cleaning span from 2.3 to 11.0 mg.m^-3^. For oven workers, levels range between 0.6 to 3.2 mg.m^-3^. The lowest levels are found for bread wrapping activities (also including slicing activities), with values lower than 1 mg.m^-3 ^[1-3,10,16,17,26-30].

Enrolment of apprentices into this study was hampered by several factors. Health is not a serious concern in this population mainly composed of teenagers, mostly from families of modest origin, not keen to contribute to health studies, according to our experience. Also, some apprentices said they would not volunteer because they feared refusal or rebuttal from their employer. Indeed, constraints posed to apprentice volunteers and to the bakery and pastry-shop managers were not trivial, in particular during Christmas or Easter holidays, when activity is great. For these reasons, we were unable to sample study locations across strata (e.g. small or large facilities; solo crafts or within commercial malls; or according to anticipated levels of concentrations of flour dust), as we had expected. Our experience, however, is that the types of facilities and indoor environments of small craft bakeries or pastries do not vary greatly in the Lorraine region. Our sample was composed of too few large facilities to draw conclusions for this category of work environment. Hence, while this is not a random sample, exposure data can be viewed as indicative of typical values currently found in small facilities. In order to assess the comparability of volunteers that underwent exposure measurements and subjects who only contributed to the questionnaire study, we compared 17 descriptive variables (e.g. type of shops, ventilation characteristics, number of working hours over the week, and different production activities such as daily number of mixing, dividing and shaping dough into pieces), both using the whole study sample and selecting only the second graders; none of these variables showed statistical differences (all p value > 0.09). One should note, however, that our results might underestimate true exposure distributions since, in our experience, employers of apprentices working in poor conditions were less likely to enrol in the study. Questionnaires were designed to obtain descriptive information on work activities and environmental conditions of apprentices in bakeries and pastries. In order to minimize recall bias, tasks we explored for a typical day to be chosen within the ongoing week; it was frequently the previous day.

Why work tasks were found to differ according to year of training among pastry apprentices but not among bakery apprentices may relate to their relative complexity. Learning how to prepare bread loafs does not take a long time while pastry making requires more technical know-how. Hence, bakery apprentices soon start to work with the baker during the first year. Bakery apprentices may also be trained to do some pastry, and conversely. Interestingly, training level differences among the two groups occur for these alternate activities, confirming that introduction to pastry know-how occurs later among bakery apprentices while, because they are more experienced, second year pastry apprentices may often help their employer to prepare bread.

Unfortunately, because it is such a routine gesture, sifting (or sieving) could not be specifically quantified with the study questionnaires; now, it incurs suspension of flour dust and therefore is an important exposing activity [11,17,28].

Comparison of exposure results across the literature should be done with caution because time and space sampling procedures, measurement techniques and methods of analyses are not readily comparable. One reason is that all published data deal with older subjects, rather than with apprentices. Working conditions of bakers or pastry-makers vary across countries according to bread or cake making processes. Further, our results are expressed in terms of personal shift exposure rather than workplace concentrations, and many published data focus on exposures during specific tasks incurring higher exposures. Finally, we measured PM_2.5 _and PM_10 _particles fractions, while literature data usually describe the 'respirable' (in the range of [1 – 4] μm), 'thoracic' ([1 – 10] μm), 'inhalable' particles ([1–100] μm) and total dust [1,2,31]. We acknowledge this may render comparisons with literature data less easy. In addition to practical reasons (availability of samplers), we wanted to compare exposure levels for two particle fractions. Also, one should keep in mind that differences between published exposure data, and how respirable and thoracic particles relate respectively to our PM_2.5 _and PM_10 _results depend on the granulometric distribution of flour, which is not documented in the literature and is most probably site and time specific, according to flour production processes. Personal exposures to total dust were evaluated in three large British bakeries, with geometric means ranging from 0.4 to 6.4 mg.m^-3 ^[3]. The same authors assessed peak total dust exposures during task measurements and described geometric means between 1.4 and 42.9 mg.m^-3 ^[8]. One French personal flour dust exposure study in an industrial bakery showed average levels about 4.9 mg.m^-3 ^[32]. Two exposure studies encompassed personal inhalable dust measurements among bakers doing three tasks: dough mixing, dough forming and oven control [26,27]. Mean dust exposure levels of dough mixing bakers were 5.5 and 7.5 mg.m^-3 ^respectively, 2.7 – 2.5 mg/m^3 ^for dough forming, and 1.2 – 3.2 mg.m^-3 ^among bakers working in the oven control area [26,27]. Burstyn *et al*. measured personal inhalable exposures to flour dust and found exposures spanning from 0.1 to 110 mg.m^-3 ^according to tasks [28]. A study separated bread bakeries (n = 19) and cake bakeries (n = 3) using personal samplers of total inhalable dust. Higher exposures were found for sieving activities (8.2 mg.m^-3^), followed by weighing (2.7 mg.m^-3^); the same tasks performed with no exhaust ventilation in the room showed greater values [11]. In another study, the geometric means of personal inhalable flour dust during sieving mixing or weighing were 4.7 mg.m^-3^, 3.3 mg.m^-3 ^for dough making activities and greater for cleaning activities (3.8 mg.m^-3^) [17].

Respiratory symptoms and sensitisation in relation to exposure of bakers to flour dust have been described in several studies [1,7,13,33-35]. In large or industrial bakeries, prevalence of nasal symptoms (19%), chest symptoms (13%) and chest tightness (7%) were associated with workplace total dust concentrations ranging about [0.01–11.0] mg.m^-3 ^[1]. In the Netherlands, a study in confectioneries, crisp-bake (rusk) factory, rye-bread factory and small bakeries, showed prevalences of rhinitis and chest tightness among workers respectively of 15% and 5%, with geometric means of full-shift airborne inhalable dust concentrations spanning from 0.6 to 3.0 mg.m^-3 ^(with range [0.1–37.7] mg.m^-3^) [9,10,13].

With cautious comparison, as stated above, one may see that our exposure figures are in general smaller than those found in the literature for inhalable or total dust. Further, exposures of apprentices are beyond the French TWA-TLV for non specific respirable and inhalable dust (5 and 10 mg.m^-3 ^respectively, with no specific limit value for flour dust) [31] and beyond the British flour dust MEL which was set at 10 mg.m^-3 ^[17,36]. Finland, Iceland and Norway have established exposure limits for total organic dust at 5 mg.m^-3 ^while in Denmark and Germany, flour dust occupational exposure limits are respectively 3 and 4 mg.m^-3 ^[37]. Now, the ACGIH recommends a lower 0.5 mg.m^-3 ^TLV for inhalable flour dust, with a view to protect against sensitization and other respiratory symptoms [37,38]. Our study PM_10 _average exposure values are close to this ACGIH TLV among pastry apprentices, and greater among bakery apprentices, a finding which is of concern despite compliance with existing standards in Europe. Exceedance also holds true among bakers for PM_2.5_.

We are not aware of exposure studies among apprentices. However, respiratory conditions and skin tests reactivity have been described among bakery trainees. An increased trend of positive skin tests was found in two surveys separated by five years in the 60ies and 70ies [18,19]. A recent study described incidence rates of respiratory symptoms (shortness of breath, wheezing, exercise induced symptoms) among pastry-makers apprentices of 5.4 per 100 person-years, 8.7 per 100 p-y and 7.7 per 100 p-y respectively [23]. Cough, dyspnoea, rhinitis, conjunctivitis and positive skin prick tests (SPT) to flour allergens were reported among bakery apprentices [20]. The authors concluded that SPT with common and occupational allergens should be performed among bakery apprentices before their training to identify subjects at highest risk of sensitisation [22,24]. In another study the same authors demonstrated hypersensitivity to occupational allergens among 18.1% of bakery apprentices, by skin prick tests, with occurrence of IgE antigen during the follow-up period. Incidence of occupational asthma and rhinitis was shown to increase with duration of exposure [21].

## Conclusion

Manipulation of flour dust is a known risk factor for respiratory conditions, in particular for asthma. While lower than the French TWA-TLV for respirable or inhalable non specific dust, personal exposure values of young bakery apprentices are greater than the ACGIH recommendation. General or process ventilation devices were not frequent in the study facilities, and this is likely to contribute to these exposure values. Further, sifting while preparing bread loafs has been shown to generate inhalation of dust. These findings suggest that further occupational hygiene progresses may result from a combination of efforts entailing modification of the granulometric profile of flour used for sifting, that would lead to more rapid deposition, improvements in facility and process ventilation, and information of bakers, including young apprentices during their training, as to potential hazards in the workplace. Also, flour dust exposures could be reduced using up-to-date process technology (i.e. decrease large sieving machinery) and proper cleaning procedures.

## List of abbreviations

PM_2.5 _= Particule matter 2.5 μm

PM_10 _= Particule matter 10 μm

## Competing interests

The author(s) declare that they have no competing interests.

## Authors' contributions

EMG coordinated the study, designed the questionnaire, conducted field investigations and performed the filters analyses and the statistical analysis. She is the main author of the manuscript. JFB contributed technical help and has given final approval of the version to be published. LM contributed to the statistical analysis. CP was an advisor for the study design and has given final approval of the version to be published. DZN directed the study and supervised writing of the manuscript. All authors read and approved the final manuscript.

## Pre-publication history

The pre-publication history for this paper can be accessed here:


